# Developmental piRNA profiles of the invasive vector mosquito *Aedes albopictus*

**DOI:** 10.1186/s13071-016-1815-8

**Published:** 2016-09-29

**Authors:** Peiwen Liu, Yunqiao Dong, Jinbao Gu, Santhosh Puthiyakunnon, Yang Wu, Xiao-Guang Chen

**Affiliations:** 1 Department of Pathogen Biology, Guangdong Provincial Key Laboratory of Tropical Disease Research, School of Public Health, Southern Medical University, Guangzhou, Guangdong 510515 China; 2 Reproductive Medical Centre of Guangdong Women and Children Hospital, Guangzhou, Guangdong 511442 China

**Keywords:** *Aedes albopictus*, Piwi-interacting RNAs, Transposable elements

## Abstract

**Background:**

In eukaryotic organisms, Piwi-interacting RNAs (piRNAs) control the activities of mobile genetic elements and ensure genome maintenance. Recent evidence indicates that piRNAs are involved in multiple biological pathways, including transcriptional regulation of protein-coding genes, sex determination and even interactions between host and pathogens. *Aedes albopictus* is a major invasive species that transmits a number of viral diseases in humans. *Ae. albopictus* has the largest genome and the highest abundance of repetitive sequences when compared with members that belong to Culicidae with a published genome. Analysis of piRNA profiles will provide a developmental and evolutionary perspective on piRNAs in *Ae. albopictus*.

**Methods:**

piRNAs were identified and characterized during the development of *Ae. albopictus*, and piRNA expression patterns in adult males and females as well as sugar-fed females and blood-fed females were compared.

**Results:**

Our results reveal that, despite the large genome size of * Ae. albopictus*, the piRNA pool of * Ae. albopictus* (1.2 × 10^7^) is smaller than those of *Aedes aegypti* (1.7 × 10^7^) and *Drosophila melanogaster* (1.6 × 10^7^). In *Ae. albopictus*, piRNAs displayed the highest abundance at the embryo stage and the lowest abundance at the pupal stage. Approximately 50 % of the piRNAs mapped to intergenic regions with no known functions. Approximately 30 % of the piRNAs mapped to repetitive elements, and 77.69 % of these repeat-derived piRNAs mapped to Class I TEs; 45.42 % of the observed piRNA reads originated from piRNA clusters, and most of the top 10 highest expressed piRNA clusters and 100 highest expressed piRNAs from each stage displayed biased expression patterns across the developmental stages. All anti-sense-derived piRNAs displayed a preference for uridine at the 5′ end; however, the sense-derived piRNAs showed adenine bias at the tenth nucleotide position and a typical ping-pong signature, suggesting that the biogenesis of piRNAs was conserved throughout development. Our results also show that 962 piRNAs displayed sex-biased expression, and 522 piRNAs showed higher expression in the blood-fed females than in the sugar-fed females.

**Conclusions:**

Our results suggest that piRNAs, aside from silencing transposable elements in *Ae. albopictus*, may have a role in other biological pathways.

**Electronic supplementary material:**

The online version of this article (doi:10.1186/s13071-016-1815-8) contains supplementary material, which is available to authorized users.

## Background

Small RNAs, which range from 19 to 30 nucleotides (nt), are ubiquitous, versatile regulatory molecules for gene expression in plants, invertebrates, vertebrates and many fungi [1, 2]. Major classes of these regulatory small RNAs include small interfering RNAs (siRNAs) and microRNAs (miRNAs), which differ with respect to their biogenesis but play important roles in post-transcriptional gene silencing [3, 4]. In addition to siRNAs and miRNAs, a third class of small non-coding RNAs, PIWI-interacting RNAs (piRNAs) has been identified in germ cells and somatic cells of vertebrates and invertebrates [5–7]. piRNA lacks clear secondary structure motifs; the length of a piRNA is between 24 and 31 nt with a strong 5′ terminal uridine or tenth position adenosine bias [8, 9].

There is growing information on the biogenesis of piRNA that are composed of the primary piRNA processing pathway and an amplification loop referred to as the ping-pong cycle. piRNAs differ from other small regulatory non-coding RNAs like (ncRNAs) such as siRNAs and miRNAs in many respects. They are processed from unidirectional single-stranded RNA precursors transcribed from intergenic repetitive elements, transposons, or large piRNA clusters and are produced independently of either Drosha or Dicer [10–12]. In *Drosophila melanogaster*, biogenesis of piRNA requires three PIWI proteins: P-element induced wimpy testis (Piwi), Aubergine (Aub) and Argonaute 3 (Ago3) [13, 14]. piRNAs are mainly generated from distinct chromosomal region that are referred to as piRNA clusters. First, the long single-stranded piRNA precursors in the sense or antisense orientation are transcribed from piRNA clusters, and then these precursor transcripts serve as the basis for piRNA production. Subsequently, the 5′ ends of precursor piRNAs might be generated by cleavage with a Phospholipase D-like protein, nuclease Zucchini (Zuc) [15–17]. These 5′ ends trimmed precursor piRNAs are then loaded onto PIWI proteins and are further trimmed from their 3′ end to the size of mature piRNAs by an unknown 3–5′ exonuclease. Finally, they are 2′-*O*-methylated at their 3′ ends with DmHen1/Pimet methyltransferase to produce mature Piwi-piRNA complexes or Piwi-piRISCs [18, 19]. Proteins associated with primary piRNAs are Piwi and Aub, and Aub- and Piwi-bound antisense piRNAs frequently begin with uridine at their 5′ ends (1-U) [20, 21]. Aub-piRISCs interact with transcripts of transposon through base pairing and eventually trigger the ping-pong cycle pathways. Then, the target transcripts are cleaved by Aub-piRISCs and the 5′- cleavage products are released and degraded, whereas the 3′- cleavage products are loaded onto Ago3 and 2′-*O*-methylated at the 3′ ends and subsequently processed into secondary piRNAs. Ago3-bound sense piRNAs are enriched for adenosine at position 10 (10-A) [22], and Aub-associated antisense and Ago3-associated sense piRNAs often overlap by precisely 10 nt from their 5′ ends [21, 23, 24].

The primary function of piRNAs is to repress transposable elements (TEs) in both the germ cell and somatic tissues, and this function is highly conserved across many animal species. Furthermore, some piRNAs are non-repetitive and non-transposon-related. A specific population of *D. melanogaster* piRNAs may be play an important role in non-repetitive, protein-coding genes regulation. Moreover, female-specific piRNA of *Bombyx mori* is a genetic switch in the WZ sex determination hierarchy. Some arbovirus infections also trigger the piRNA pathway in mosquito cells, suggesting that piRNAs may play additional unknown roles [25–27].

The Asian tiger mosquito (*Aedes albopictus*) is an epidemiologically important vector that transmits many viral infections, such as yellow fever, dengue and Chikungunya [28]. The genome of *Ae. albopictus* has relatively abundant repetitive sequences, which comprise 71 % of the genome (the highest composition of all sequenced mosquito species). This high-repeat content can easily explain the larger genome size than that of *Ae. aegypti*, a member of the same subgenus (*Stegomyia*) and the only other mosquito species with a sequenced genome larger than 1 Gigabase pairs (Gbp) [29]. Here, we explored small-RNA profiles of *Ae. albopictus* to determine how piRNA clusters are organized and how the piRNA expression profile changes during development (especially piRNAs related to sex bias that are differentially expressed in blood-fed adult females).

## Methods

### Mosquitoes

The Foshan (Guangdong, China) strain of *Ae. albopictus* originated in Foshan, Guangdong Province, PRC, and it was established in the laboratory in 1981. All mosquitoes were maintained in humidified incubators at 25 ± 1 °C on a 12-h light:dark photocycle.

### Construction and sequencing of a small RNA library

Embryos were collected 0–24 h after egg deposition by using a damp collection cup. Larval samples were collected at each instar stage and combined. Pupal samples were collected from a pool of specimens with varied ages. Male and female adults were collected 5 days post-emergence. Three-day-old adult females were fed mouse blood, collected 2 days after feeding, and pooled. Total RNA was extracted using TRIzol® reagent (Invitrogen, Life Technologies, Carlsbad, USA). Small RNAs were purified using polyacrylamide gel electrophoresis to enrich molecules in the range of 18–30 nt and are sequentially ligated to 5′- (5′-GUU CAG AGU UCU ACA GUC CGA CGA UC-3′) and 3′-end RNA oligonucleotide adaptors (5′-pUC GUA UGC CGU CUU CUG CUU GUidT-3′) using T4 RNA ligase. cDNA libraries were constructed using oligo(dT) primer by SuperScript III Reverse Transcriptase (Invitrogen, Carlsbad, CA) followed by 18 cycles of polymerase chain reaction (PCR) amplification. Purified PCR products were used directly for cluster generation and sequenced with the Illumina Genome Analyzer (Illumina, San Diego, USA). All sequencing was performed by the Beijing Genomics Institute (BGI), Shenzhen. Raw sequence reads were submitted to the National Center for Biotechnology Information (NCBI) short-read archive (Accession number: SRA060684).

### Data analyses

Adaptor sequences were removed, and low-quality tags were cleaned. Contamination due to adaptor-adaptor ligation was removed using Trimmomatic-0.30 with default settings [30]. Unique reads of 24–30 nt were selected for further analysis and mapped to the *Ae. albopictus* genome (Accession ID: JXUM00000000) by using Short Oligonucleotide Analysis Package 2 (SOAP2) [31]. Reads mapped to rRNAs, tRNAs, snRNAs, snoRNAs, miRNAs and exons were excluded; reads containing poly-A/T/C/G nucleotides (minimum of 8 homopolymer repeat nucleotides) were also removed. The rest of the genome that matched with small RNAs was used as piRNA-like small RNAs for further analysis.

Heat-map was generated with hierarchical clustering analysis by MeV 4.8 software (https://sourceforge.net/projects/mev-tm4/files/mev-tm4/). The images of sequence logos were created using the R package seqLogo [32]. The piRNAs sequence pool size in *Ae. albopictus* was estimated based on the observed number of piRNAs in each library and on the amount of overlap between libraries using the formula described previously [33, 34].

### Ping-pong signature

Ping-pong pairs were defined as precise 10-nt 5′-end overlaps between sense and antisense piRNAs [35]. To determine the fraction of piRNAs in ping-pong pairs, we counted all uniquely mapped piRNA reads and plotted the distance between the 5′ ends of complementary small RNAs by using a previously described method [34].

### piRNA clusters

All PIWI reads with perfect matches to the *Ae. albopictus* genome were normalized to total genome mapping reads for comparison. The approach was similar to that used by Arensburge et al. [34]. A total of 405,464 *Ae. albopictus* supercontigs were individually scanned using a 5-kb sliding window, and windows with 10 or more piRNA sequences mapped to them were identified. The identified windows were merged if they were less than 20 kb apart. Boundaries of putative cluster loci were detected by scanning for the location of the furthest piRNA sequence on either end of a locus. Cluster expression density of each sample was normalized to the size of the library, and the resulting data were expressed in tags per million (TPM). A methodological difference was that, we used PIWI reads of at least 200 supporting reads across 6 libraries for PIWI cluster identification to minimize false-positive noise.

### Enrichment analysis of gene ontology (GO) functions and gene pathways

The DAVID functional annotation tool (http://david.abcc.ncifcrf.gov/) was used to perform GO classification and pathway annotation of piRNA-generating mRNAs [36]. Functional annotation terms from the ontologies of “biological processes” and “molecular function” with an EASE threshold of 0.1 and a count threshold of 2 were recorded [37]. Enrichment score cutoff was set to 1. Genes that generate piRNAs were assigned to pathways by using the online DAVID tool [36].

## Results

### Sequence analysis of small RNAs

We sequenced 6 small RNA libraries for the sequential developmental stages: embryos, larvae, pupae, adult males, sugar-fed adult females and blood-fed *Ae. albopictus* females to analyze temporal, sex bias and blood meal-induced expression profiles of small RNAs. A total of 101,354,117 reads were sequenced. After removing 5′-adaptors, trimming 3′-adaptor sequences, and filtering out low-quality reads, 80,959,738 reads were obtained (Additional file 1: Table S1). Then, reads longer than 18 nt were counted for further analysis. A total of 72,330,525 reads that were more than 18 nt in length were obtained, representing 10,330,130 unique tags (Additional file 1: Table S1).

**Fig. 1:**
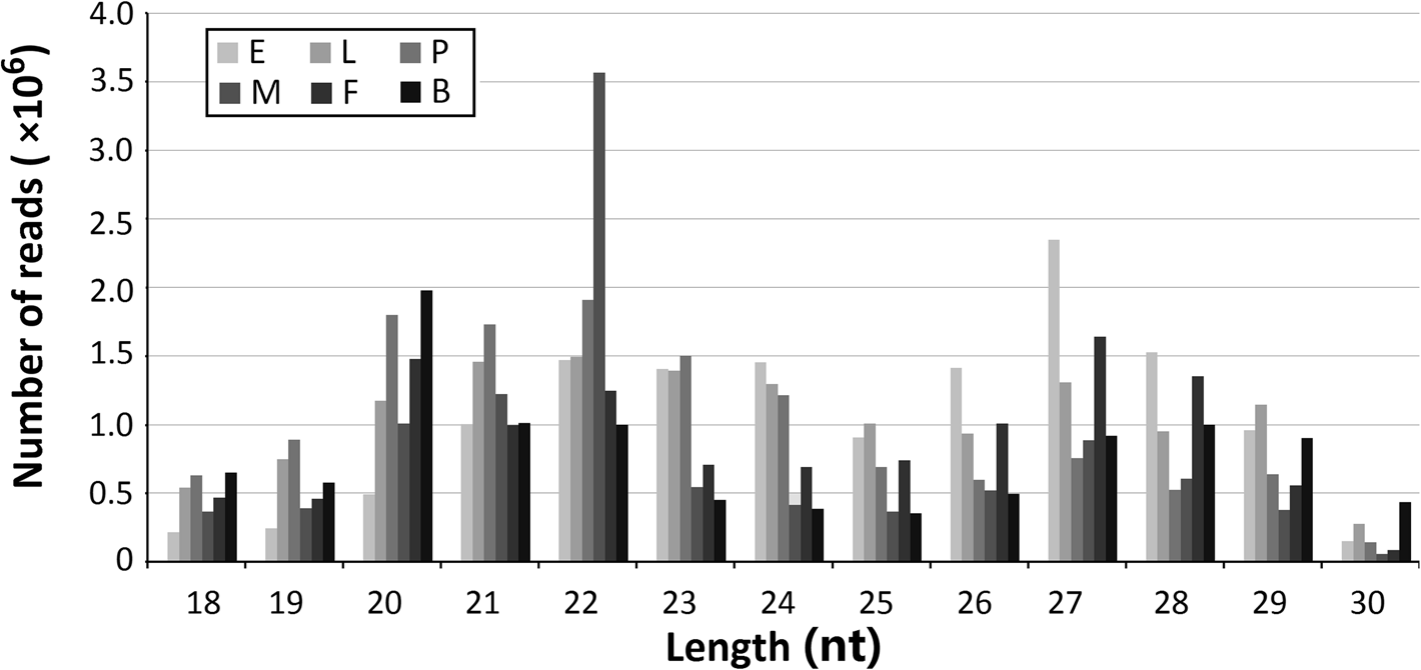
The nucleotide length distribution of sequence tags obtained from the six *Aedes albopictus* small RNA (sRNA) libraries. Size distribution and relative frequency in each sample are shown for the small RNAs derived from embryos, larvae, pupae, adult males, sugar-fed adult females, and blood-fed adult females. *Abbreviations*: nt, length of small RNA read in nucleotides; S, sample from which the small RNAs were sequenced; E, embryos; L, larvae; P, pupae; M, adult males; F, adult females and B, blood-fed adult females

In the larvae, pupae, adult male and blood-fed female samples, the major peak occurred at 20–23 nt, followed by a minor peak at 26–28 nt; in the embryo and sugar-fed female samples, the secondary peak occurred at 20–23 nt, and the primary peak shifted to 26–28 nt (Fig. 1). piRNA peaks at all stages displayed a Gaussian distribution; however, the blood-fed adult females exhibited a slight difference, with a 27–28 nt peak instead of a 26–27 nt peak as in the other stages.

### Prediction of piRNAs

Reads corresponding to piRNAs were first identified by excluding unique reads of 24–30 nt that mapped to rRNAs, tRNAs, snRNAs, snoRNAs and miRNAs and reads that contained poly-A/T/C/G nucleotides and then mapping the rest of the 24–30 nt reads to the *Ae. albopictus* genome. As a result, a total of 68,940,526 reads were filtered out and 12,019,212 reads were identified; 4,425,402 reads had a unique match in the *Ae. albopictus* genome, and 7,593,810 reads were mapped in multiple positions (Additional file 2: Table S2). The size of *Ae. albopictus* piRNA pool was estimated to 1.2 × 10^7^ (minimum estimate 8.9 × 10^6^, maximum estimate 1.7 × 10^7^) using the methodology previously defined (see Methods). Compared to the previously reported piRNA pools of *Ae. aegypti* (1.7 × 10^7^) and *D. melanogaster* (1.6 × 10^7^), the piRNA pool of *Ae. albopictus* is smaller. The predicted piRNAs were assembled in 6 samples and had signature piRNA characteristics, including a preference for uridine at the 5′ end (75.81 %), which is a main characteristic of primary piRNAs, and an A-bias at the 10th nucleotide position (40.61 %), which suggests that piRNAs are primarily produced by the ping-pong cycle [38]. The similar nucleotide preference was observed across the 6 analyzed libraries (Additional file 3: Figure S1). Finally, we analyzed the ping-pong signature of all libraries and the characteristic signal of 10-nt sense-antisense 5′-overlaps between individual piRNAs. At all stages, the overlapping piRNAs exhibited a classic ping-pong signature (Additional file 4: Figure S2).

**Fig. 2:**
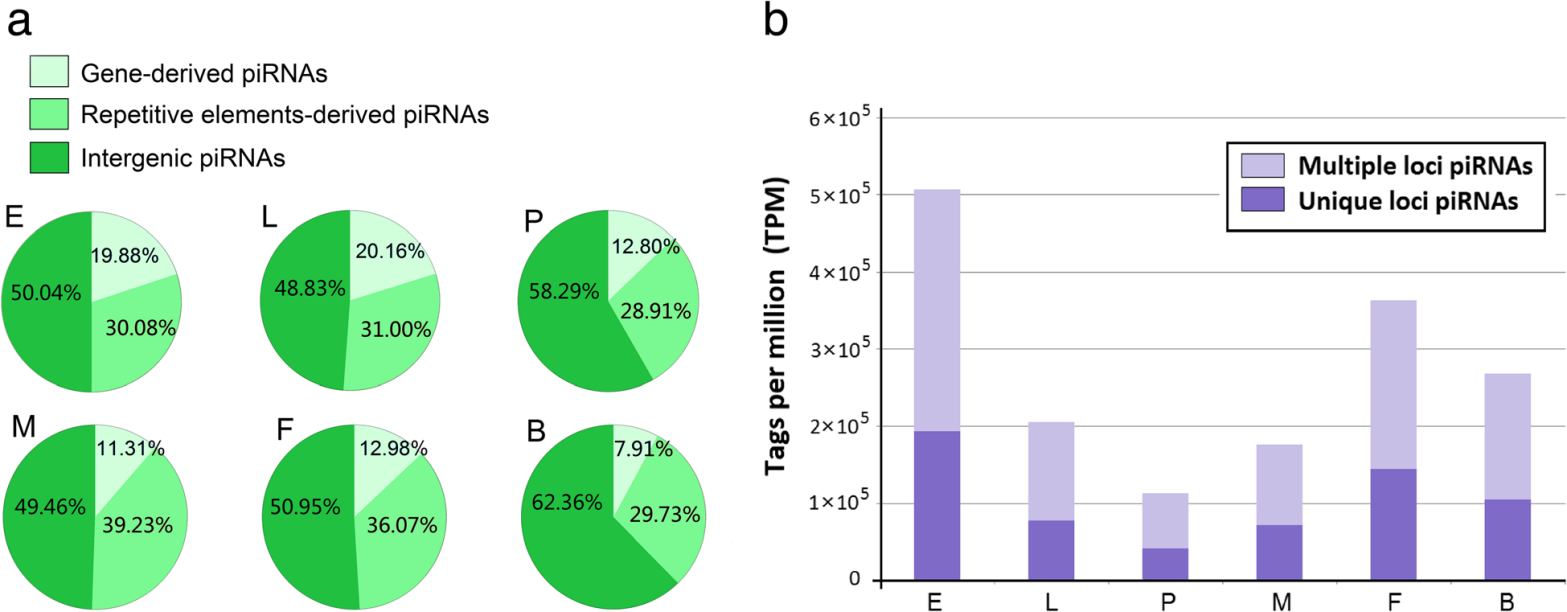
piRNA-like small RNAs in six developmental stages of the *Aedes albopictus* dataset. **a** The proportions of gene-derived piRNA (*light green*), repetitive element-derived piRNA (*green*), and intergenic piRNA reads (*deep green*) in a six *Aedes albopictus* small RNA (sRNA) dataset. **b** The abundance of piRNAs in six developmental stages of *Aedes albopictus*. piRNAs mapped to unique genomic positions (*deep purple*) and piRNAs mapped to multiple genomic positions (*purple*). *Abbreviations*: M, adult male; F, adult female; L, larvae; E, embryos; B, blood-fed female

piRNAs that mapped uniquely to repetitive elements, mRNAs, and intergenic regions were regarded as repetitive element-derived piRNAs (RTPRs), gene-derived piRNAs and intergenic piRNAs, respectively. Approximately 50 % of the piRNAs mapped to intergenic regions with no known functions, followed by reads mapped to RTPRs (approximately 30 %; Fig. 2a). We studied the piRNA expression profile during the life span of *Ae. albopictus* and observed that piRNAs are most abundant in the embryos (Fig. 2b).

### Repeat-derived piRNAs

**Fig. 3:**
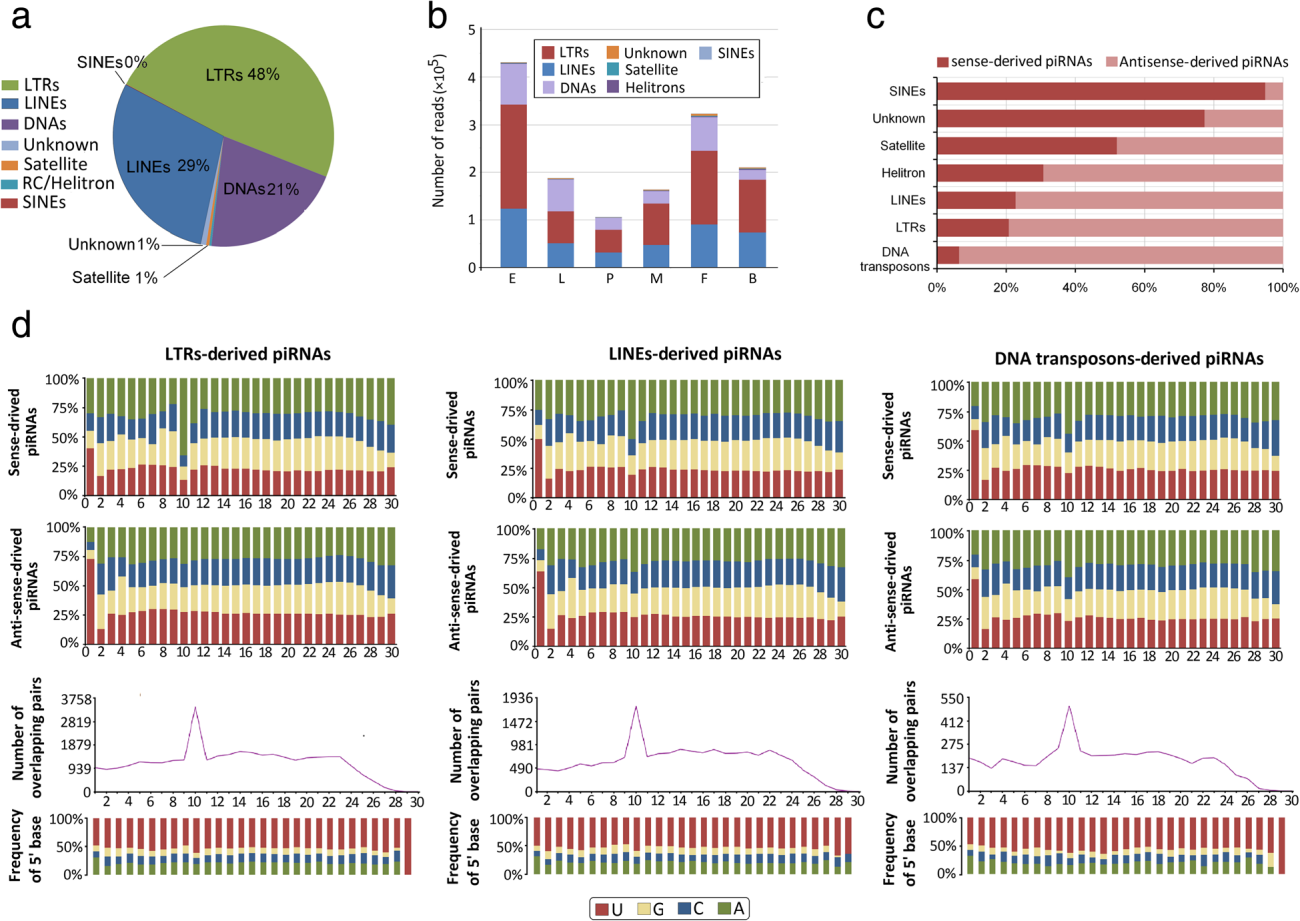
Characterization of repeat-derived piRNAs. **a** The proportion of piRNAs mapped to repeat sequences shows that major piRNAs are preferentially produced from LTR, LINE, and DNA transposons within the transposon group. **b** Abundance and distribution of repeat-derived piRNAs in various developmental stages; repeat-derived piRNAs displayed similar compositions. **c** Strand preference of repeat-derived piRNAs. **d** Upper panel: base composition of repeat-derived piRNAs of three major TE sequences, LTR, LINE, and DNA transposons. The X-axis represents the nucleotide position relative to the 5′ ends of the piRNAs. The Y-axis represents the percentage of base bias. Lower pane: ping-pong pair analysis of three major TE sequences, LTR, LINE, and DNA transposons. The length of overlap is shown on the horizontal axes. Indicated above each axis is the number of possible overlapping pairs of small RNAs with the specified overlap size. Indicated below each axis is the relative frequency of the 5′ base identity for overlapping sequences. The colour code for the bases is indicated in the centre box

Repression of transposable elements is considered as the primary function of piRNAs; thus, we mapped piRNAs to annotated *Ae. albopictus* transposons [29]. Across the 6 analyzed libraries, repeat-derived piRNAs constituted 32.13 % of the total reads of uniquely mapped piRNAs. Most (77.69 %) of the repeat piRNAs mapped to Class I TEs (retrotransposons), including 62.06 %, 37.83 % and 0.1 % repeat piRNAs mapped to long interspersed nuclear elements (LINEs), long terminal repeats (LTRs) and small interspersed nuclear elements (SINEs), respectively. Only a fraction of the transposon-specific piRNAs (20.75 %) mapped to class II TEs (DNA transposons). Proportionally, few piRNAs mapped to tandem repeat satellite DNAs (0.44 %) and helitrons (0.33 %) [39] (Fig. 3a; Additional file 5: Table S3). At all developmental stages, repeat-derived piRNAs displayed a similar composition (Fig. 3b; Additional file 5: Table S3). Strand preference was observed for piRNA mapped to repeats; the sense/antisense ratio differed considerably for the transposon families LINEs (0.29), SINEs (18.31), LTR (0.26), DNA transposons (0.07) and helitrons (0.44) (Fig. 3c; Additional file 5: Table S3).

For evidence of ongoing TE repression *via* the ping-pong cycle, we analyzed the 5′ overlaps of sense and antisense piRNAs mapped to 3 major TE sequences (LTRs, LINEs, and DNA transposons). We observed a marked ping-pong signature for all TE-related piRNAs, indicating PIWI-dependent processing (Fig. 3d). Furthermore, antisense piRNAs show a strong 1U bias (73.36 %, 63.77 % and 58.56 %, respectively), and typical elevation for 10A (65.52 %, 50.09 % and 44.14 %, respectively) can be observed for sense piRNAs reads in LTRs, LINEs and DNA transposons (Fig. 3d).

### Gene-derived piRNAs

**Fig. 4:**
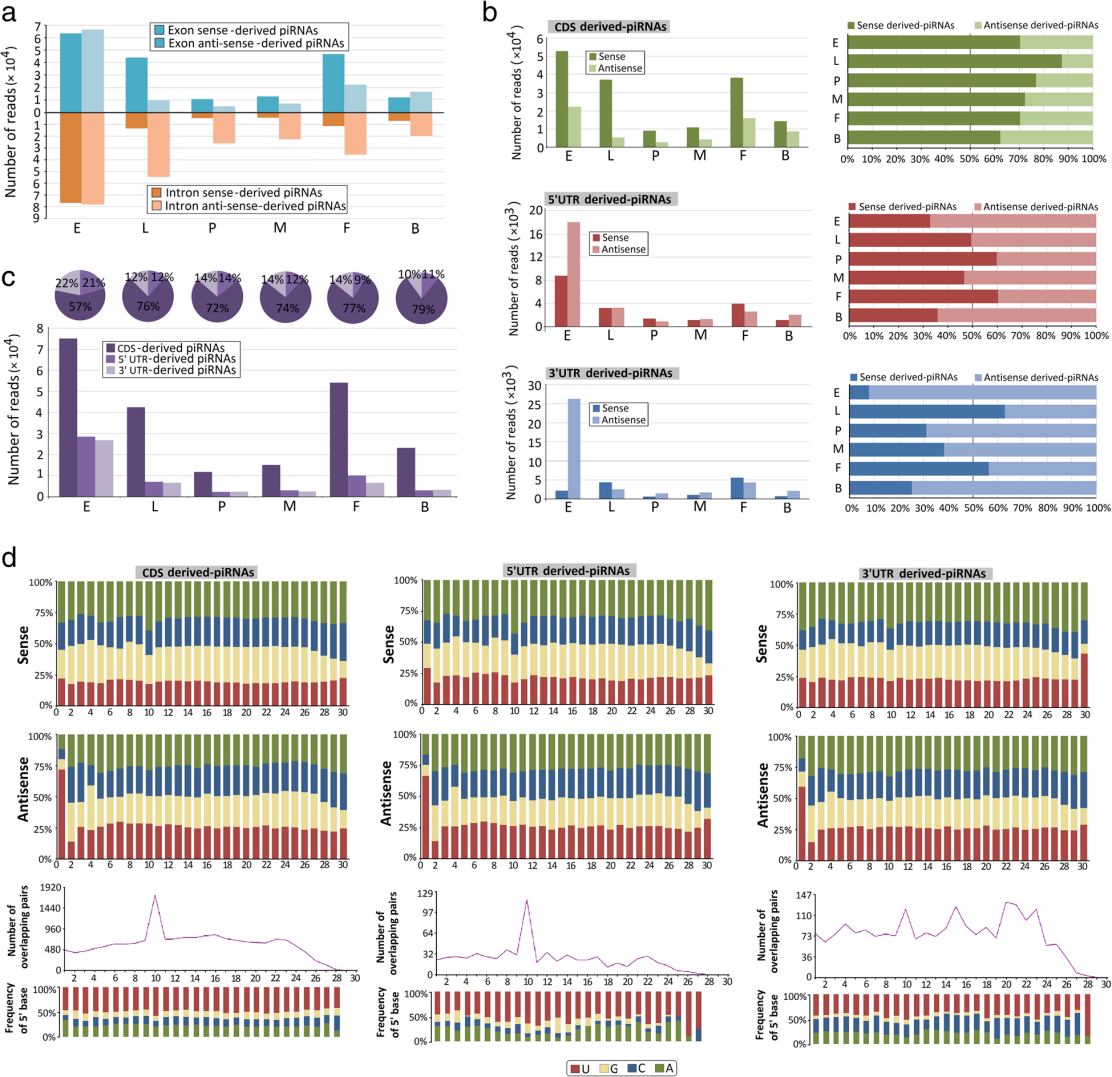
Characterization of gene-derived piRNAs. **a** Abundances and strand preference of exon-derived and intron-derived piRNAs from six developmental stages. **b** Abundance and percentage of CDSs, 5′ UTR- and 3′ UTR-derived piRNAs. **c** Abundance and strand preference of CDS-derived reads, 5′ UTR-derived reads and 3′ UTR-derived reads from six developmental stages of the *Ae. albopictus* dataset. **d** Upper panel: Base composition of CDSs, 5′ UTR- and 3′ UTR-derived piRNAs. The X-axis represents the nucleotide position relative to the 5′ ends of the piRNAs. The Y-axis represents the percentage of base bias. Lower pane: ping-pong pair analysis of CDSs, 5′ UTR- and 3′ UTR-derived piRNAs. The length of overlap is shown on the horizontal axes. Indicated above each axis is the number of possible overlapping pairs of small RNAs with a specified overlap size. Indicated below each axis is the relative frequency of the 5′ base identity for overlapping sequences. The colour code for bases is indicated in the centre box

To analyze the potential impact of piRNA functions on protein-coding genes, the mapped piRNA reads were initially screened for sequences mapped to annotated protein-coding genes. piRNAs mapped to genes constituted 15.22 % of all uniquely mapped piRNAs. In the 6 assembled libraries, 52.65 % of the reads mapped to introns and 47.35 % mapped to exons of mRNAs. Intriguingly, with respect to protein-coding genes, we found that most piRNA reads mapped to intronic sequences in the sense orientation in all developmental libraries, which cannot be explained by the processing of spliced mRNAs. Furthermore, major mapping to exonic regions was observed in the antisense orientation, with the exception of the embryo and blood-fed female adult stages (Fig. 4a; Additional file 6: Table S4). Reads mapped to exons preferentially mapped to coding sequences (CDS; 68.58 %), followed by 3′ UTRs (16.61 %) and 5′ UTRs (14.81 %) (Fig. 4b; Additional file 6: Table S4). CDS-derived piRNAs preferentially arose from the sense strand, suggesting that they are generated from precursor mRNA (pre-mRNA; Fig. 4c) [40]. piRNAs mapped to 5′ UTRs and 3′ UTRs, but they did not show consistent strand bias on the basis of developmental stage, although, they mostly preferred an antisense orientation (Fig. 4c).

Antisense exon-derived piRNAs (including CDS-, 5′ UTR- and 3′ UTR-derived piRNAs) typically have 1-U (71.81 %, 66.19 %, and 59.00 %, respectively), and only sense CDS- and 5′ UTR-derived piRNAs exhibit a marginal preference for 10-A (39.81 % and 43.13 %, respectively; Fig. 4d). Of the 3′ UTR-derived piRNAs, most mapped to the antisense strand and maintained 5′ 1-U preference but lacked a strong 10A bias (32.69 %) and ping-pong signature (Fig. 4d). piRNAs that mapped to the intron region displayed antisense bias and 5′ 1-U preference but lacked strong 10A bias and ping-pong components in nearly every developmental library (Additional file 7: Figure S3).

### GO analysis of gene-derived piRNAs

To identify potentially conserved functions of gene-derived piRNAs, we used a method that relies on collected transcript IDs and generated gene-originating piRNAs from 15,666 genes. Enrichment of functional annotation terms (FATs) for these unique genes was performed using GO terms and Kyoto Encyclopedia of Genes and Genomes (KEGG) pathway annotation and DAVID gene annotation tool (http://david.abcc.ncifcrf.gov/). FATs with enrichment scores > 1 was regarded as enriched (Additional file 8: Table S5). A similar GO distribution was observed in piRNAs mapped to genes across the 6 analyzed libraries (Additional file 9: Figure S4). A number of piRNA-generating genes were involved in cellular processes, metabolic processes, cell, cell part, DNA binding and catalytic activities, indicating that these functions may be associated with metabolism and accelerated growth and development of *Ae. albopictus* [41, 42]. KEGG gene pathway analysis showed that piRNA-generating genes were involved in 270 pathways, primarily protein processing in the endoplasmic reticulum, glycolysis/gluconeogenesis, endocytosis and fatty acid metabolism (Additional file 8: Table S5).

Generally, piRNAs map to specific mRNA transcripts in a very similar pattern to TE transcripts with distinct signs of ping-pong cycle mediated amplification; this implies that mRNAs are not only subjected to primary biogenesis pathway but can also be targeted by primary piRNAs and processed into antisense secondary piRNAs [43].

### Chromosomal distribution and piRNA gene clusters

Millions of individual piRNAs can be mapped to a few hundred discrete genomic loci called piRNA clusters. These piRNA clusters usually range from 20 to 100 kb and are the sources of most piRNAs [20]. To analyze piRNA clusters in the *Ae. albopictus* genome, we used the approach described by Arensburger [34]. In each library, the piRNAs with more than 200 reads were selected for the cluster analysis. In total, 1,577 piRNAs were mapped to only 1 location, and 2,844 piRNAs hit more than 2 genomic locations. A total of 643 piRNA clusters, which are up to 10 kb in length, were identified (Additional file 10: Table S6). The identified clusters could potentially generate 45.42 % of the observed piRNA reads. These piRNA clusters are widely distributed throughout the genome and cover nearly 2 Mb. Each cluster contains 10–697 distinguished piRNAs (average, 24.96 piRNAs). A total of 325 and 304 piRNA clusters mapped only on the minus or plus strands, respectively (Additional file 10: Table S6). A total of 13 clusters were distributed on 2 strands but in a divergent, non-overlapping manner (Additional file 10: Table S6). We also searched for the repetitive elements and coding genes within the piRNA clusters. Our results showed that 164 clusters contain at least one TE, whereas only 43 clusters contain coding genes (Additional file 10: Table S6).

**Fig. 5:**
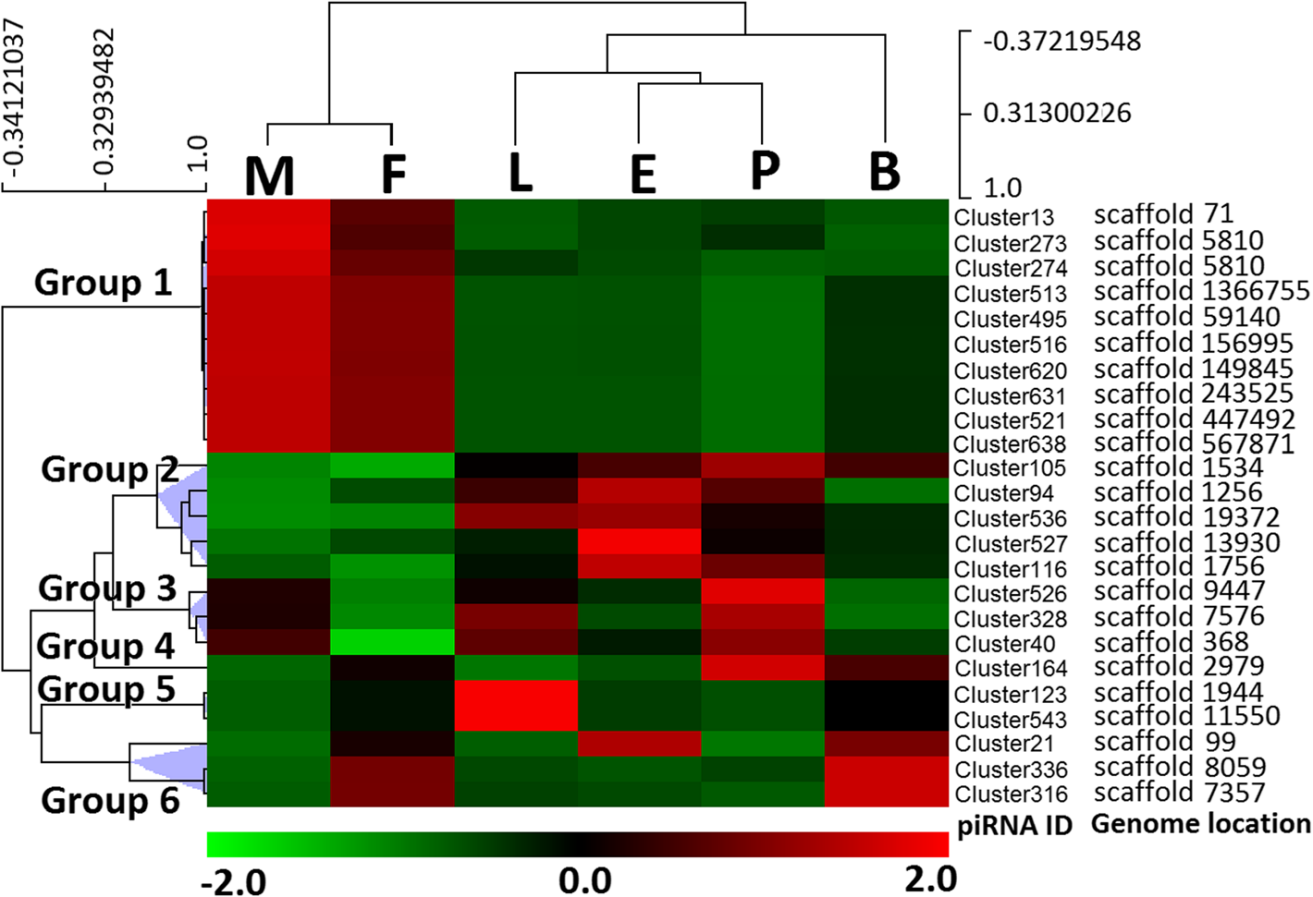
Hierarchical grouping of 24 piRNA clusters. Normalized expression profiles of 24 piRNA clusters from six developmental stages were grouped. The stages are in columns, and the piRNA clusters are in rows. Red indicates that a piRNA cluster is strongly represented at the stage, whereas green indicates weak representation. piRNA clusters with similar expression patterns group together. There are six groups (1–6) with variable numbers of sub-groups. *Abbreviations*: E, embryos; L, larvae; P, pupae; M, adult males; F, adult females; B, blood-fed adult females

Locations of the piRNA clusters on the *Ae. albopictus* supercontigs were broadly consistent in all libraries; however, these clusters differed with respect to transcript abundance. Therefore, we focused on the top 10 piRNA clusters in each library; for each stage, the top 10 piRNA clusters cover no more than 10 kb of the genome and account for half of the total cluster reads. After assembling the top 10 piRNA clusters in the 6 libraries, a total of 24 unique clusters were selected for further analysis; among these clusters, only 2 maintained the top 10 transcript abundance at all stages, and the broad developmental expression patterns of 24 clusters were revealed *via* cluster analysis on the basis of TPM. Six expression patterns were identified (Fig. 5; Additional file 11: Table S7). Hierarchical clustering showed that the piRNA clusters in group 1 had high expression in the adults. Group 2 had high expression in the non-adult stages; groups 2 and 4 tend to be highly expressed in the pupae. Group 5 was larva-specific, and group 6 was expressed at high levels in both the sugar- and blood-fed females. The piRNA clusters were considered to be derived from long, single-stranded piRNA precursors with no apparent secondary structure [44]. *Ae. albopictus* piRNA clusters also exhibited extreme strand bias; the 24 clusters described above had piRNAs mapped predominantly to 1 strand.

### Developmental profiling of *Ae. albopictus* piRNAs

**Fig. 6:**
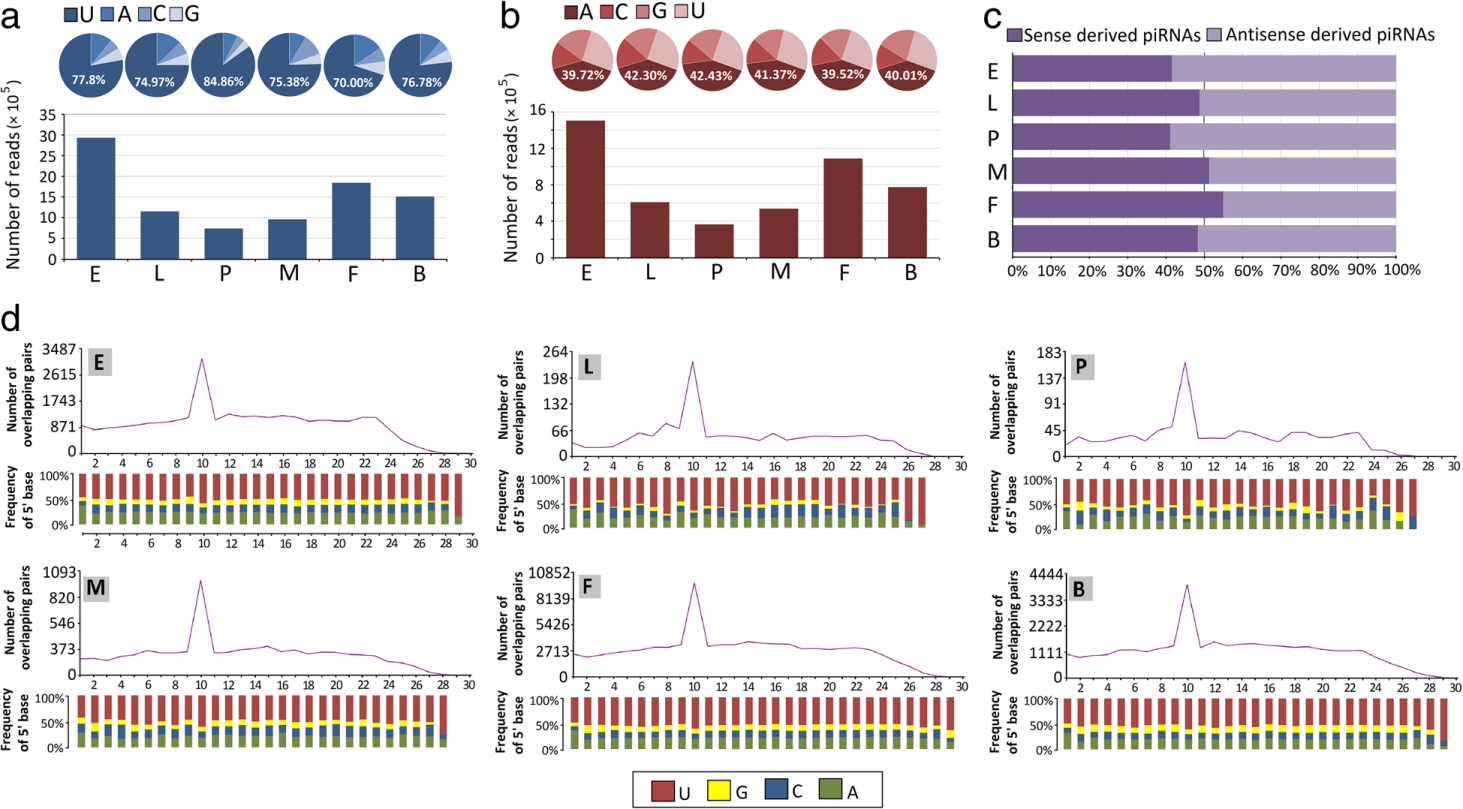
Developmental characterization of piRNAs. **a** Ratios (*upper panel*) and abundances (*lower panel*) of piRNAs with uridine at their 5′ ends in various developmental stage libraries. **b** Ratios (*upper panel*) and abundances (*lower panel*) of piRNAs with adenosine at position 10 in various developmental stage libraries. **c** Strand bias in various developmental stage libraries. **d** Ping-pong signature of piRNAs in various developmental stage libraries

Primary piRNAs, defined by a preference for uridine at the 5′ end [45], were observed in the embryo library; similar percentages were obtained for the other libraries (Fig. 6a). Secondary piRNAs generated by a ping-pong mechanism and identified by an adenine bias at the 10^th^ nucleotide position [46] showed decreased abundance from the embryo to pupa stages and increased abundance in the adult stages (Fig. 6b). Furthermore, a ping-pong signature was nearly consistent across the developmental stages, suggesting that the biogenesis of piRNAs was conserved throughout the development despite shifts in 5′ 1-U and 10-A piRNA expression levels (Fig. 6d). Strand bias was observed in piRNAs at various developmental stages. piRNAs at adult stages, with the exception of blood-fed females, showed a slight sense bias; the other stages displayed antisense bias (Fig. 6c). To explore the developmental profile of distinctly derived piRNAs, we further analyzed the repeat-derived and gene-derived piRNAs, respectively. As a result, both repeat-derived and gene-derived piRNAs showed typical 5′ 1-U bias and 10-A piRNA across all developmental stages, whereas the repeat-derived piRNA displayed higher 5′ 1-U percentage but lower 10-A percentage than that of gene-derived piRNAs (Additional file 12: Figure S5a). Strand bias analysis displayed completely reverse strand origination between repeat-derived and gene-derived piRNAs, however both repeat-derived and gene-derived piRNAs showed relatively conserved strand bias across developmental stages, respectively (Additional file 12: Figure S5b). Among gene-derived piRNAs, except adults stage, piRNAs showed only a weak ping-pong signature, but for repeat-derived piRNAs, all stages displayed typical ping-pong signature except the larvae stage (Additional file 12: Figure S5c).

**Fig. 7:**
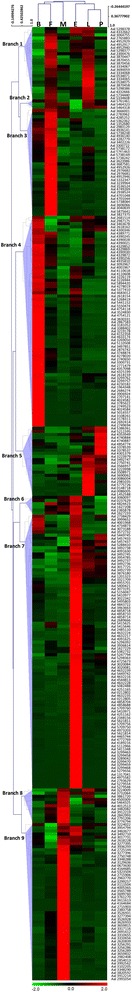
Hierarchical clustering of piRNA expression. Normalized expression profiles of 303 piRNAs from six developmental stages were clustered. The stages are in columns, and the piRNAs are in rows. Red indicates that a gene is strongly represented at the stage, black indicates the intermediate expression whereas green indicates low expression. The colour scale bar shown at the bottom of the figure indicates the log_2_ value of fold change. piRNAs with similar expression patterns cluster together. There are nine clusters with variable numbers of sub-clusters. *Abbreviations*: E, embryos; L, larvae; P, pupae; M, adult males; F, adult females; B, blood-fed adult females

Hundred most abundant piRNAs from each stage were collected; 303 total piRNAs were evaluated on the basis of normalized read counts per piRNA (Additional file 13: Table S8; Fig. 7), and 9 expression patterns were identified (9 major branches in dendrogram). Hierarchical clustering showed that most of the piRNAs had stage-specific high expression. Embryo-specific piRNA accumulation in the largest branch 7 [30] supported the hypothesis that piRNAs may be critical for early development. A few piRNAs in branch 5 are highly expressed in larvae or pupae, whereas piRNAs in branch 3 and 9 are female and male adult-specific, respectively. Blood-feeding also tend to induce the expression of some specific piRNAs that assembled in branch 4.

### Sex-biased piRNAs and differentially expressed piRNAs in the sugar-fed and blood-fed females

**Fig. 8:**
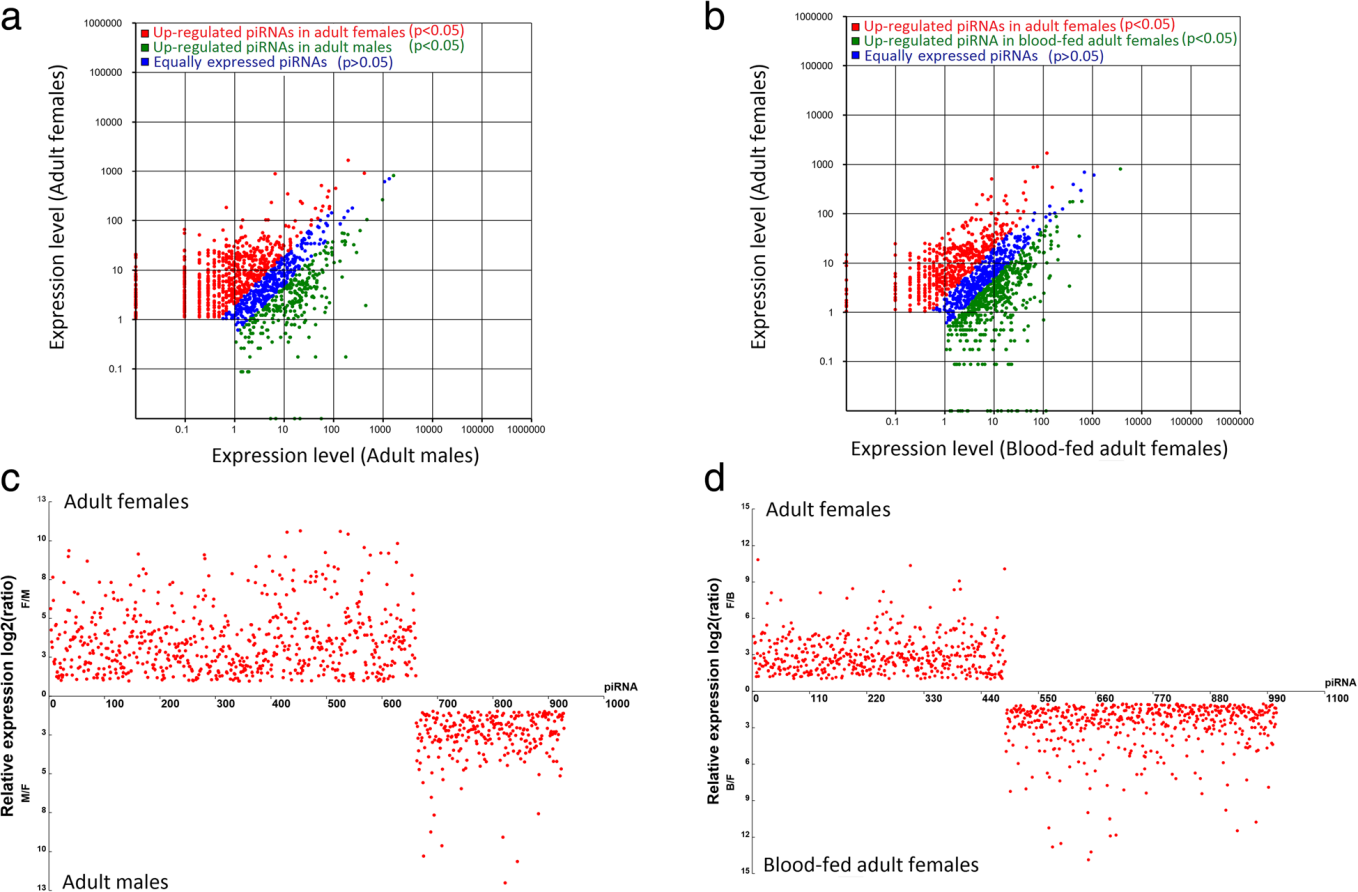
Pairwise comparison results of the expression levels of piRNAs in the three libraries. Scatter plot of up- and downregulated piRNAs in **a** adult males and adult females and **b** sugar-fed adult females and blood-fed adult females. Each point represents a piRNA. The X axis and Y axis show the expression levels of piRNAs in two libraries. The red points indicate the more highly expressed piRNAs in the library on the X axis (adjusted *P* < 0.05), the blue points represent equally expressed piRNAs (adjusted *P* > 0.05), and the green points represent more highly expressed piRNAs in the library on the Y axis (adjusted *P* < 0.05). The relative expression levels of various piRNAs in **c** adult males and adult females and **d** sugar-fed adult females and blood-fed adult females. The X axis indicates the ranking of piRNA ID number of either 659 female-bias or 303 male-bias expression piRNAs (Additional file 13: Table S9) in **c**, whereas 522 blood-fed females higher expression piRNAs and 485 sugar-fed females higher expression piRNAs (Additional file 15: Table S11) in **d**; the Y axis shows the relative expression level (log2 ratio)

To determine differences in piRNA expression between sexes, we analyzed the expression profiles of 4-day-old sugar-fed adult females and males. In total, 962 piRNAs displayed sex-biased expression (*P* ≤ 0.05, log_2_ ratio ≥ 1; Fig. 8; Additional file 14: Table S9). Of these piRNAs, 659 and 303 transcripts showed significantly higher abundance in the females and males, respectively. Furthermore, these sex-biased piRNAs showed bias towards different annotated types of genomic loci. piRNAs that exhibited relatively high levels of expression in the males were primarily derived from the intergenic region and repeat sequence LTR/Gypsy; piRNAs that exhibited higher expression in the females than in the males were mapped to intergenic regions, LTR/Gypsy, LTR, and LINE/R1 (Additional file 14: Table S9 and Additional file 15: Table S10). Comparison of piRNA patterns in the sugar-fed and blood-fed females showed that 1,006 piRNAs were differentially expressed (Poisson distribution, *P* < 0.05); 522 piRNAs showed higher expression in the blood-fed females than in the sugar-fed females (Additional file 16: Table S11; Fig. 7). Furthermore, 18 piRNAs with no reads were found in the sugar-fed females and were thought to be specific to blood-fed females. These upregulated piRNAs in the blood-fed females also showed a bias towards annotated types of genomic loci; they were primarily derived from intergenic regions, LTR/Gypsy, and DNA transposons (Additional file 17: Table S12).

## Discussion

The prominent function of the piRNA pathway is to silence the re-replication and transposition of TEs [12]. Recently, extensive researches on the piRNA pathway have advanced our understanding of the relationships between TEs and the host genome, and the important role of TEs in genetic, biological and developmental processes [47–49]. TEs comprise a major proportion of the genomes of most arthropods: up to 47 % of the genome of *Ae. aegypti*, approximately 17 % of *Anopheles gambiae*, 29 % of *Culex quinquefasciatus* and 20 % of *Drosophila* [50]. piRNA pathway components mutants cause significant over-replication of transposons, and overactive transposon mobilization could be the cause of DNA mutations [51–53]. Notably, mutations can occur when transposon insertion or homologous recombination between inserted TEs, and may increase genetic variation, which results in selection and evolutionary changes. *Ae. albopictus* is one of the 100 most destructive invasive species on Earth [54]. *Ae. albopictus* exhibits great adaptability to a broad spectrum of environmental conditions, is phenotypically polymorphic, and displays variation in its vectorial capacity to mosquito-borne viruses (MBV) [28, 55, 56]; it has a large genome (1,967 Megabase pairs, Mbp) and is rich in repetitive DNA and TEs that comprise as much as 61 % of the genome. However, considering the large genome and higher transposon content, the size of the piRNA pool in *Ae. albopictus* (1.2 × 10^7^) is smaller than that of the piRNA pools of *Ae. aegypti* (1.7 × 10^7^) and *D. melanogaster* (1.6 × 10^7^). This will help us to understand how TEs and piRNAs are implicated in changes to gene expressions that enable *Ae. albopictus* to be a successful invasive species [57, 58], since TEs play an important role in the responsive capacity of their hosts in the face of environmental challenges [59, 60], piRNA pathway can be regarded as the key to the protection of the genome against the activity of TEs.

The genome of *Ae. albopictus* contains all major groups of TEs; LINE, LTR, DNA and SINE representing 34.67 %, 16.21 %, 8.52 % and 0.07 % of the entire *Ae. albopictus* genome, respectively. However, LTR-derived piRNAs show the highest expression level for TE-derived piRNAs and are similar to piRNAs observed in humans [61]. LINE-, LTR- and DNA-associated piRNAs are primarily derived from the antisense strand. The opposite trend is observed for SINEs and satellite DNAs. Our results suggest that the antisense piRNAs may recognize LTR transcripts. LTR and LINE elements contain an internal promoter for RNA polymerase II. Both of their own genes and of adjacent genes can accurately transcribe from their insertion sites [62, 63]. piRNAs may be involved in repressing LINE and LTR transcripts and preventing the expression of adjacent host genes. However, reduced abundance of piRNAs is associated with satellite DNA, since the satellite DNA-derived small RNAs were most likely generated by endogenous siRNA and guide specific heterochromatin modifications through RNA interference mechanism (RNAi) [64]. SINE-derived piRNAs display high sense bias. However, no typical 10-A or ping-pong signals were identified in any library (Additional file 18: Figure S6). Similar results were obtained in mice, indicating that the piRNA defense system does not appear to regulate SINEs in *Ae. albopictus* [65].

In contrast to *D. melanogaster* and zebrafish [6, 20], most piRNAs in *Ae. albopictus* map to repetitive regions. Most *Ae. albopictus* piRNAs are derived from intergenic regions, which are similar to those previously observed in mammals. Most intergenic piRNAs are also generated from genomic piRNA clusters. There is limited information on the functions of piRNAs derived from intergenic regions. Some piRNAs appear to be involved in the regulation of mRNAs or transposons in early embryos and gonads [66, 67]. Our results showed that the sex-biased piRNAs and highly expressed piRNAs in the sugar-fed adult females were primarily derived from an intergenic region; this finding leads us to propose a potential role for intergenic piRNAs in transposon control in gonads and early embryos.

The mechanism underlying gene-derived piRNA biogenesis has not yet been clarified. Notably, genic piRNAs are in the sense orientation relative to the direction of gene transcription and typically derived from exons in the libraries for every developmental stage, with the exception of embryos (Fig. 4a), suggesting that mature mRNA molecules are the substrate molecules for processing [68]. We observed that piRNAs preferentially mapped to CDS and then to 5′ UTRs and lastly to 3′ UTRs at each developmental stage; however, CDS show different strand bias. Notably, embryo CDS-derived and UTR-derived piRNAs show opposite strand bias. For sense-biased CDS-derived piRNAs, some of the resultant mRNA molecules may be enrolled in the piRNA biogenesis hierarchy to be processed into the observed piRNAs [68]. The 3′ UTRs of an extensive series of mRNAs were shown to be involved in piRNAs biogenesis in inmurine testes, *Drosophila* ovaries, and *Xenopus* eggs [69]. Comprehensive analysis of small RNA-seq data from serials mutants and PIWI complexes immunoprecipitates revealed that biogenesis of the 3′ UTRs derived piRNAs depends on primary piRNA elements but not on piRNA ping-pong processing (4P) components [69]. This finding is consistent with our results; we also found a deficiency in classic ping-pong signals and stronger 10A-bias in *Ae. albopictus* 3′ UTR-derived piRNAs (Fig. 4d), indicating that they originate from primary piRNA biogenesis pathways. We also detected piRNAs from the intron region with a higher antisense bias (Fig. 4a), and the lack of a classic ping-pong signal indicated that the intronic piRNAs originate from primary piRNA biogenesis pathways. Therefore, primary piRNAs appear to be produced both prior to and after transcript splicing [70]. It couldn’t exclude the possibility that 3′ UTRs isoforms resulting from alternative splicing of transcripts that generate 3′ UTR-derived piRNAs. However, accumulation of 3′ UTR-specific isoforms was not detected for these genes. On the other hand, insertion of an exogenous gene (green fluorescent protein, GFP) into 3′ UTR region of *traffic jam* (*tj*) gene, whose 3′ UTR region generates abundant piRNAs, induce production of novel piRNAs derived from gfp sequences. Furthermore, *piwi* mutants resulted in upregulation of Tj protein levels in Drosophila. These characteristics might reflect competition between the primary piRNA biogenesis machinery and mRNA translation machinery, and cannot eliminate the possibility that the primary piRNA pathways act independently of their mRNA transcripts.

We identified a number of piRNA clusters that generated nearly half (45.42 %) of the total piRNA reads. Most clusters displayed profound strand preference, with reads originated from only single-stranded RNA precursors within a cluster (uni-strand cluster). The top ten piRNA clusters in each library were assembled, and a total of 24 clusters were used to analyze developmental expression patterns. Among these clusters, ten displayed high expression in adults, especially in male adults; 7/10 of these clusters originate from the antisense strand. Many of highly expressed piRNA clusters also contained TEs sequences. The PIWI pathway is necessary for male reproductive capacity in *Drosophila* and mice [71, 72] and was thought to silence retrotransposons during postnatal spermiogenesis. Loss-of-function mutants of piwi gene will influence several distinct stages of spermatogenesis [72].

We observed variations in piRNA abundance at various developmental stages. In addition to total piRNA abundance, abundance of TE-derived, gene-derived, 5′ 1-U, and 10-A piRNAs (Fig. 3, 4 and 6) all showed similar developmental trends, and both sense- and antisense-derived piRNAs were most abundant in the embryo stage of *Ae. albopictus*. The increase in the antisense piRNA pool in embryos could be speculated to primarily originate from maternally deposited piRNAs, these maternally inherited piRNAs deposited into maturing oocyte and early embryo, and are immediately protected against TE overexpression and mobilization [73–78]. In contrast, an extensive abundance of sense derived piRNAs mainly relies on the zygotic transcription of sense transposon, a theoretical target of maternally deposited antisense piRNAs [35]. Interestingly, we also noticed an increased abundance of piRNAs in the sugar-fed adult females when compared with the blood-fed females. A similar pattern for piRNAs was previously described in *An. gambiae* [79, 80].

Nix, a key male sex-determining factor, has been identified in *Ae. aegypti*; however, mechanisms underlying sex determination and differentiation in mosquitoes are largely unknown [81]. Additional factors that involved in sex determination system of insects need to be identified. Sex-biased piRNAs in sex-specific genital organs has recently been reported in *Drosophila* [82, 83]. Sex determination in *B. mori* has been found to directly depend on sex-specific piRNA that derived from feminizing gene (Fem) in the putative female-determining region of the W chromosome [84]. This finding is the first example of a piRNA that mediates the sex determination process. We also found sex-biased piRNAs in *Ae. albopictus*, and these sex-biased piRNAs are primarily derived from repeat elements, as reported for *Drosophila* and zebrafish [82, 83]. It seems that piRNAs do not act as the primary sex determination signal in *Aedes*, considering we failed to find the *Nix-*derived piRNAs both in *Ae. aegypti* and *Ae. albopictus* (unpublished data), however we cannot exclude the possibility that sex-biased piRNA may be involved in downstream part of sex-determination hierarchy. We also found that blood-feeding induced higher expression and de novo production of piRNAs, which has also been observed in *An. gambiae* [79]; these piRNAs generally map to repeat sequences and DNA transposons, indicating that they are likely to be involved in oogenesis after blood-feeding.

## Conclusions

In conclusion, our study showed that the piRNA pool of *Ae. albopictus* is smaller than those of *Ae. aegypti* and *D. melanogaster*, although it has a larger genome. We observed variations in piRNA abundance at various developmental stages, and the abundance of TE-derived, gene-derived, 5′ 1-U, and 10-A piRNAs showed similar developmental trends. We also found biased expression of piRNAs in blood-fed adult females when compared with sugar-fed and sex-biased piRNAs. This result suggests that piRNAs, aside from silencing transposable elements in *Ae. albopictus*, may play a role in other biological pathways.

## Additional files

Additional file 1: Table S1. Small RNA sequence statistics for the six developmental stage libraries of *Aedes albopictus.* (XLS 33 kb) Additional file 2: Table S2. Small RNA sequence statistics for 24–30 nt reads matched to the *Aedes albopictus* genome assembly. (XLS 32 kb) Additional file 3: Figure S1. Sequence bias of piRNAs in the six developmental stage libraries of *Aedes albopictus*. Sequence logo was obtained using the R package seqLogo. *Abbreviations*: E, embryos; L, larvae; P, pupae; M, adult males; F, adult females and B, blood-fed adult females. (TIF 3449 kb) Additional file 4: Figure S2. Ping-pong pair analysis of intron-derived piRNAs in six developmental stages of the *Aedes albopictus* dataset. The length of overlap is shown on the horizontal axes, and indicated above each axis is the number of possible overlapping pairs of small RNAs within a specified overlap size. Vertical bars below each axis show the relative frequency of the 5′ base identity for overlapping sequences. The colour code for bases is indicated in the centre box. (TIF 637 kb) Additional file 5: Table S3. List of repeat-derived piRNAs. (XLS 33 kb) Additional file 6: Table S4. List of Gene-Derived piRNAs. (XLS 33 kb) Additional file 7: Figure S3. Characterization of intron-derived piRNAs in six developmental stages of the *Aedes albopictus* dataset. Base composition of intron-derived piRNAs. The X-axis represents the nucleotide position relative to the 5′ ends of the piRNAs. The Y-axis represents the percentage of base bias. Lower pane: ping-pong pair analysis. The length of overlap is shown on the horizontal axes. Indicated above each axis is the number of possible overlapping pairs of small RNAs within a specified overlap size. Indicated below each axis is the relative frequency of the 5′ base identity for overlapping sequences. The colour code for bases is indicated in the centre box. (TIF 2556 kb) Additional file 8: Table S5. KEGG pathway analysis of piRNA-generating genes. (XLS 340 kb) Additional file 9: Figure S4. GO classification of piRNAs mapping to genes across the six analysed libraries. The results are summarized in three main categories: biological processes, cellular components and molecular functions. (TIF 1249 kb) Additional file 10: Table S6. The cluster distribution of piRNAs in the *Aedes albopictus* genome. (XLS 268 kb) Additional file 11: Table S7. The top 10 abundance of transcription of piRNAs clusters from each developmental library. (XLS 39 kb) Additional file 12: Figure S5. Characterization of gene and repetitive elements-derived piRNAs. a Ratios of piRNAs with uridine at their 5′ ends (upper panel) and piRNAs with adenosine at position 10 (lower panel) in various developmental stage libraries. b Ratios and abundances (lower panel) of in various developmental stage libraries. b Strand bias in various developmental stage libraries. Abundance and percentage of CDSs, 5′ UTR- and 3′ UTR-derived piRNAs. c Ping-pong pair analysis of gene and repetitive elements-derived piRNAs. The length of overlap is shown on the horizontal axes. Indicated above each axis is the number of possible overlapping pairs of small RNAs with a specified overlap size. Indicated below each axis is the relative frequency of the 5′ base identity for overlapping sequences. The colour code for bases is indicated in the centre box. (TIF 4512 kb) Additional file 13: Table S8. Expression profiles of assembly top 100 piRNA profiles in six stage library. (XLSX 50 kb) Additional file 14: Table S9. Candidates of sex-bias expression piRNAs. (XLS 207 kb) Additional file 15: Table S10. Sex-bias piRNA loci in the genome. (XLS 40 kb) Additional file 16: Table S11. Candidates of upregulated piRNAs in blood-fed adult female. (XLS 209 kb) Additional file 17: Table S12. Upregulated piRNAs loci of blood-fed adult female in the genome. (XLSX 13 kb) Additional file 18: Figure S6. Characterization of SINE-derived piRNAs. Base composition of SINE-derived piRNAs. The X-axis represents the nucleotide position relative to the 5′ ends of the piRNAs. The Y-axis represents the percentage of base bias. Lower pane: ping-pong pair analysis. The length of overlap is shown on the horizontal axes. Indicated above each axis is the number of possible overlapping pairs of small RNAs within a specified overlap size. Indicated below each axis is the relative frequency of the 5′ base identity for overlapping sequences. The colour code for bases is indicated in the centre box. (TIF 730 kb)

## Abbreviations

Ago3: Argonaute 3
Aub: Aubergine
CDS: Coding sequences
FATs: Functional annotation terms
Gbp: Gigabase pairs
GFP: Green fluorescent protein
GFP: Green fluorescent protein
GO: Gene ontology
LINEs: Long interspersed nuclear elements
LTRs: Long terminal repeats
MBV: Mosquito-borne viruses
miRNAs: microRNAs
NCBI: National Center for Biotechnology Information
ncRNAs: Non-coding RNAs
nt: Nucleotides
PCR: Polymerase chain reaction
piRNAs: Piwi-interacting RNAs
Piwi: P-element induced wimpy testis
RNAi: RNA interference
RTPRs: Repetitive element-derived piRNAs
SINEs: Small interspersed nuclear elements
siRNAs: Small interfering RNAs
TEs: Transposable elements
*tj*: *Traffic jam*
TPM: Tags per million
Zuc: Zucchini
