# Supplementary figures and images for: Developmental piRNA profiles of the invasive vector mosquito Aedes albopictus
Source: Parasit Vectors. 2016 Sep 29;9:524. doi: 10.1186/s13071-016-1815-8 (PMC5041409; doi:10.1186/s13071-016-1815-8)

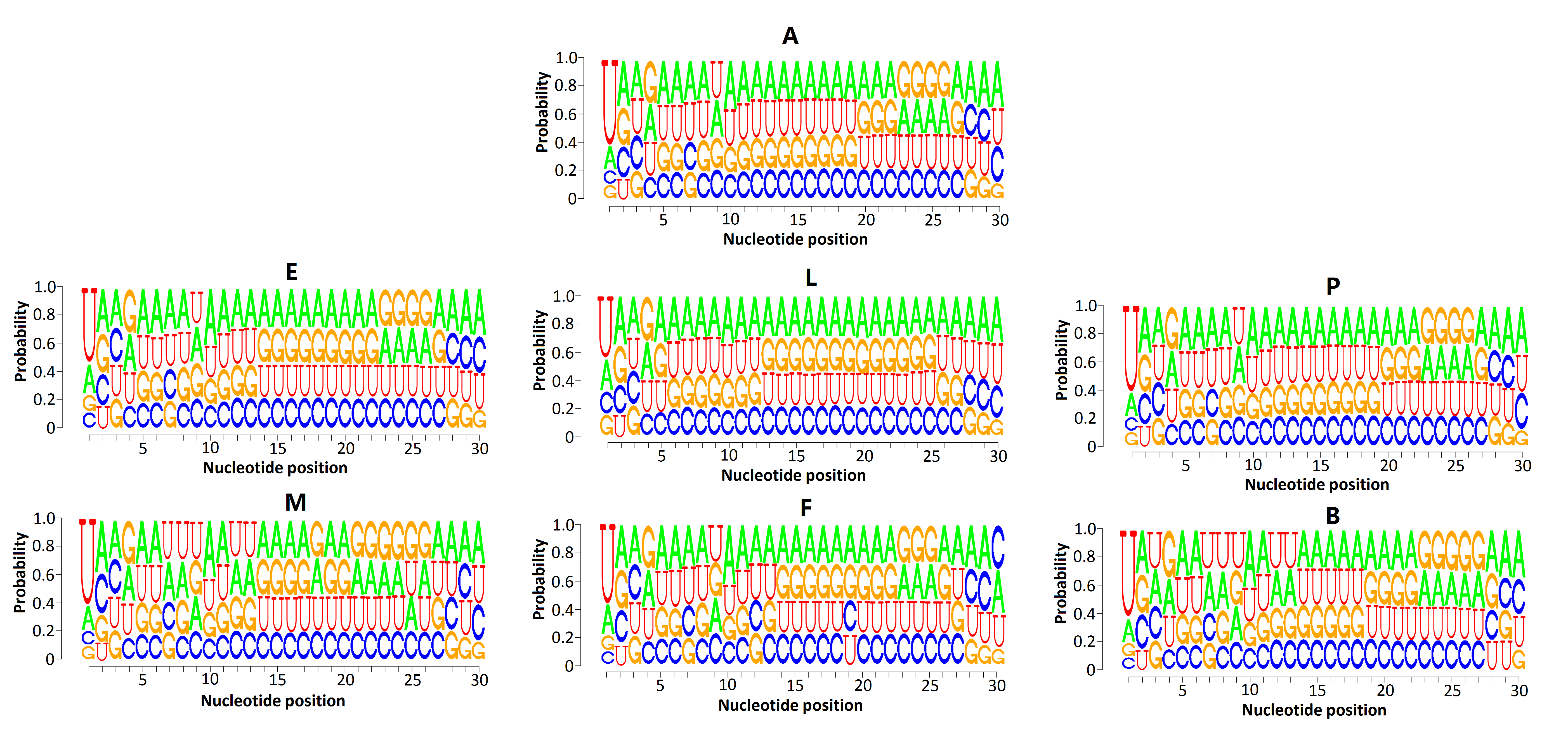

Supplement: Additional file 3: Figure S1. — Sequence bias of piRNAs in the six developmental stage libraries of Aedes albopictus. Sequence logo was obtained using the R package seqLogo. Abbreviations: E, embryos; L, larvae; P, pupae; M, adult males; F, adult females and B, blood-fed adult females. (TIF 3449 kb) [file 13071_2016_1815_MOESM3_ESM.tif]

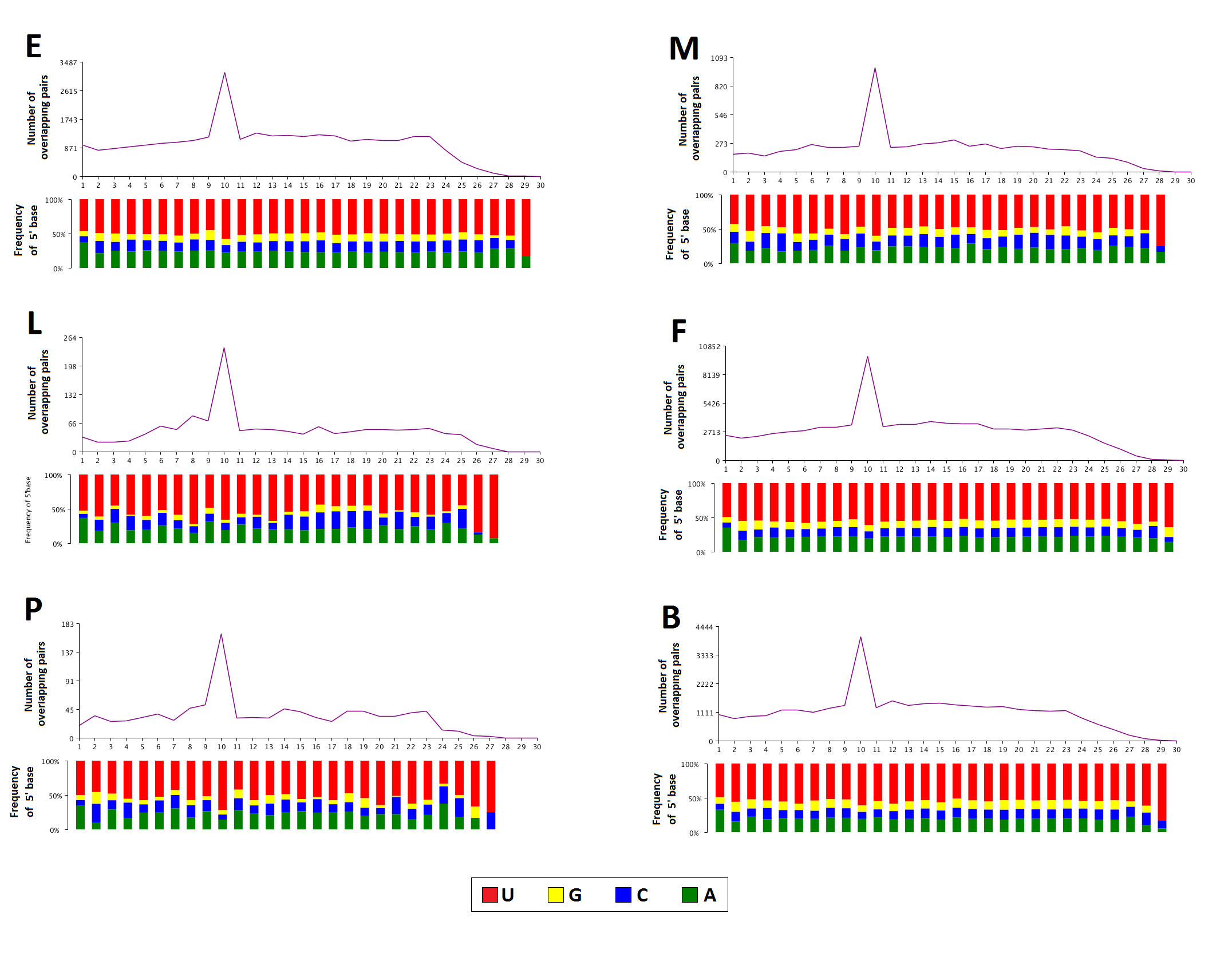

Supplement: Additional file 4: Figure S2. — Ping-pong pair analysis of intron-derived piRNAs in six developmental stages of the Aedes albopictus dataset. The length of overlap is shown on the horizontal axes, and indicated above each axis is the number of possible overlapping pairs of small RNAs within a specified overlap size. Vertical bars below each axis show the relative frequency of the 5′ base identity for overlapping sequences. The colour code for bases is indicated in the centre box. (TIF 637 kb) [file 13071_2016_1815_MOESM4_ESM.tif]

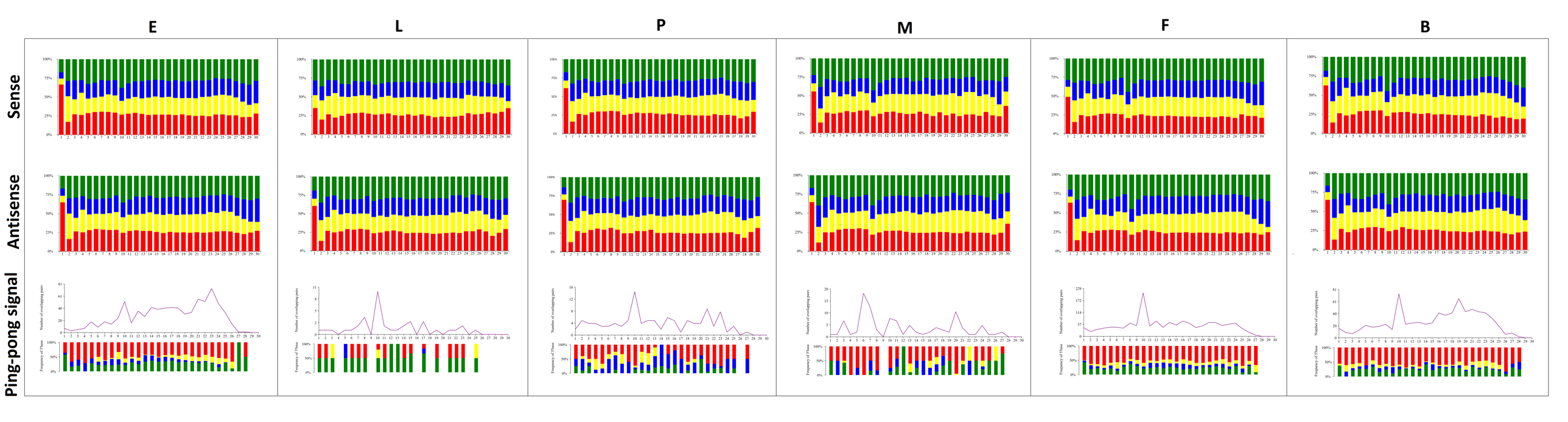

Supplement: Additional file 7: Figure S3. — Characterization of intron-derived piRNAs in six developmental stages of the Aedes albopictus dataset. Base composition of intron-derived piRNAs. The X-axis represents the nucleotide position relative to the 5′ ends of the piRNAs. The Y-axis represents the percentage of base bias. Lower pane: ping-pong pair analysis. The length of overlap is shown on the horizontal axes. Indicated above each axis is the number of possible overlapping pairs of small RNAs within a specified overlap size. Indicated below each axis is the relative frequency of the 5′ base identity for overlapping sequences. The colour code for bases is indicated in the centre box. (TIF 2556 kb) [file 13071_2016_1815_MOESM7_ESM.tif]

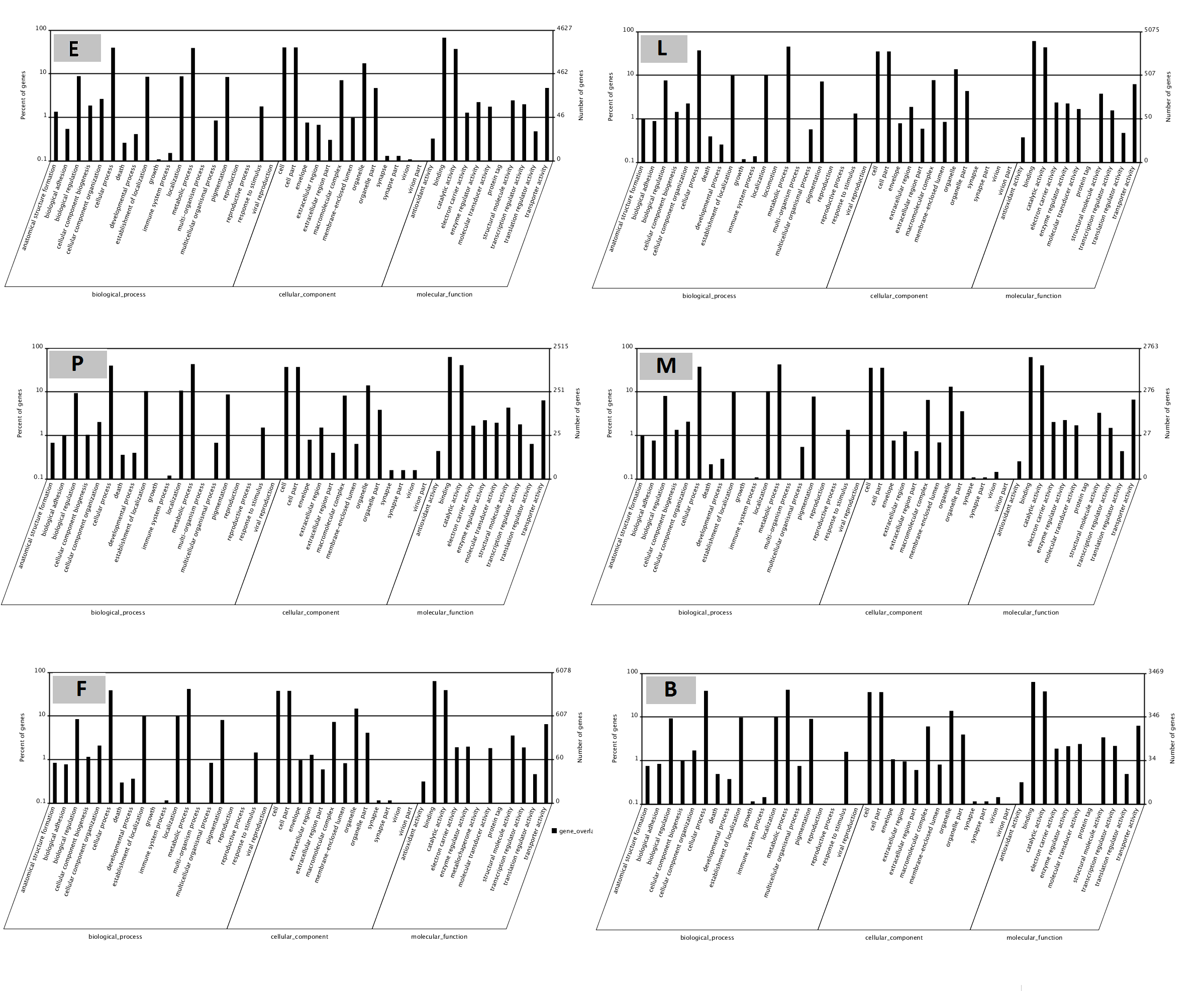

Supplement: Additional file 9: Figure S4. — GO classification of piRNAs mapping to genes across the six analysed libraries. The results are summarized in three main categories: biological processes, cellular components and molecular functions. (TIF 1249 kb) [file 13071_2016_1815_MOESM9_ESM.tif]

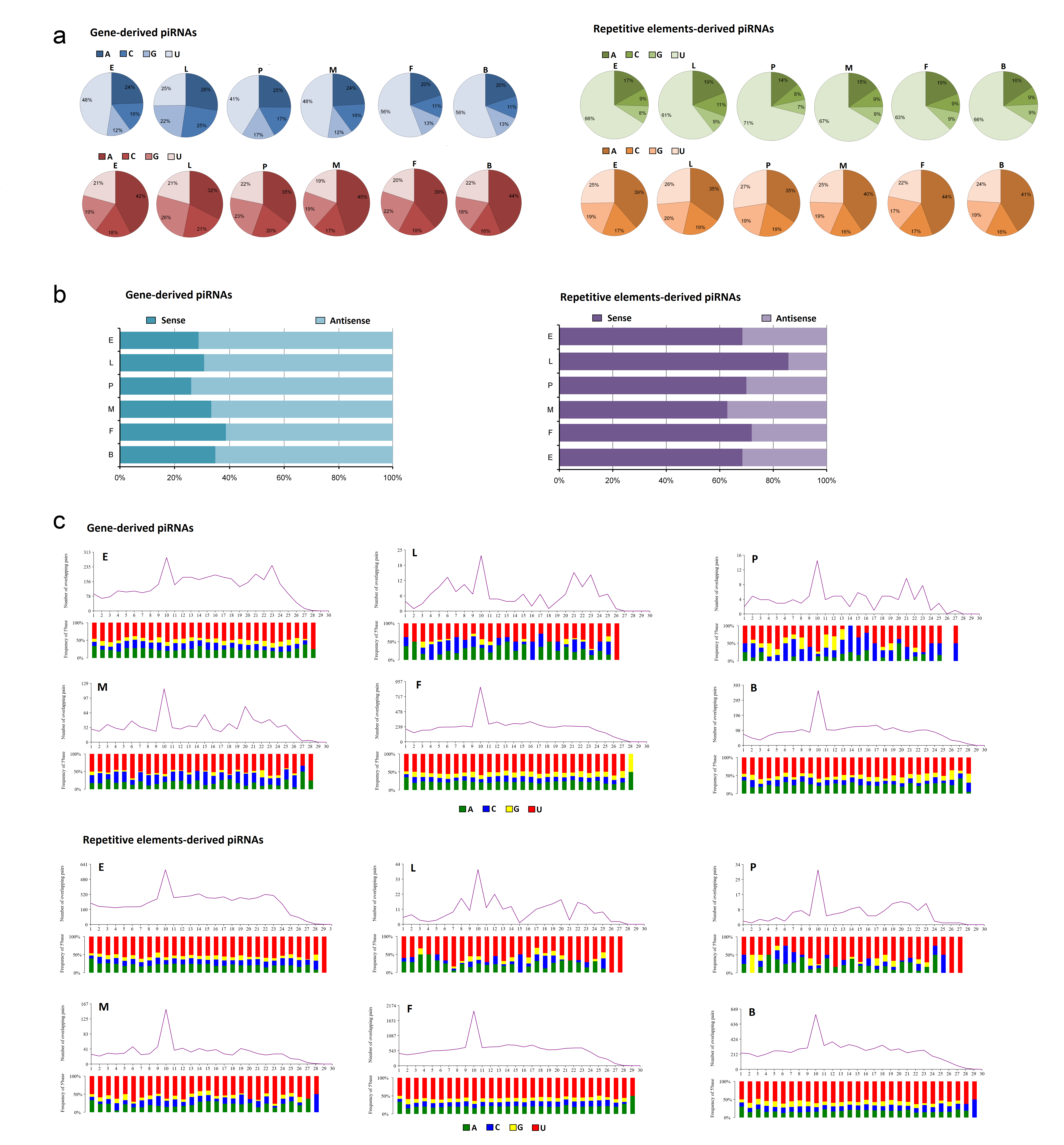

Supplement: Additional file 12: Figure S5. — Characterization of gene and repetitive elements-derived piRNAs. a Ratios of piRNAs with uridine at their 5′ ends (upper panel) and piRNAs with adenosine at position 10 (lower panel) in various developmental stage libraries. b Ratios and abundances (lower panel) of in various developmental stage libraries. b Strand bias in various developmental stage libraries. Abundance and percentage of CDSs, 5′ UTR- and 3′ UTR-derived piRNAs. c Ping-pong pair analysis of gene and repetitive elements-derived piRNAs. The length of overlap is shown on the horizontal axes. Indicated above each axis is the number of possible overlapping pairs of small RNAs with a specified overlap size. Indicated below each axis is the relative frequency of the 5′ base identity for overlapping sequences. The colour code for bases is indicated in the centre box. (TIF 4512 kb) [file 13071_2016_1815_MOESM12_ESM.tif]

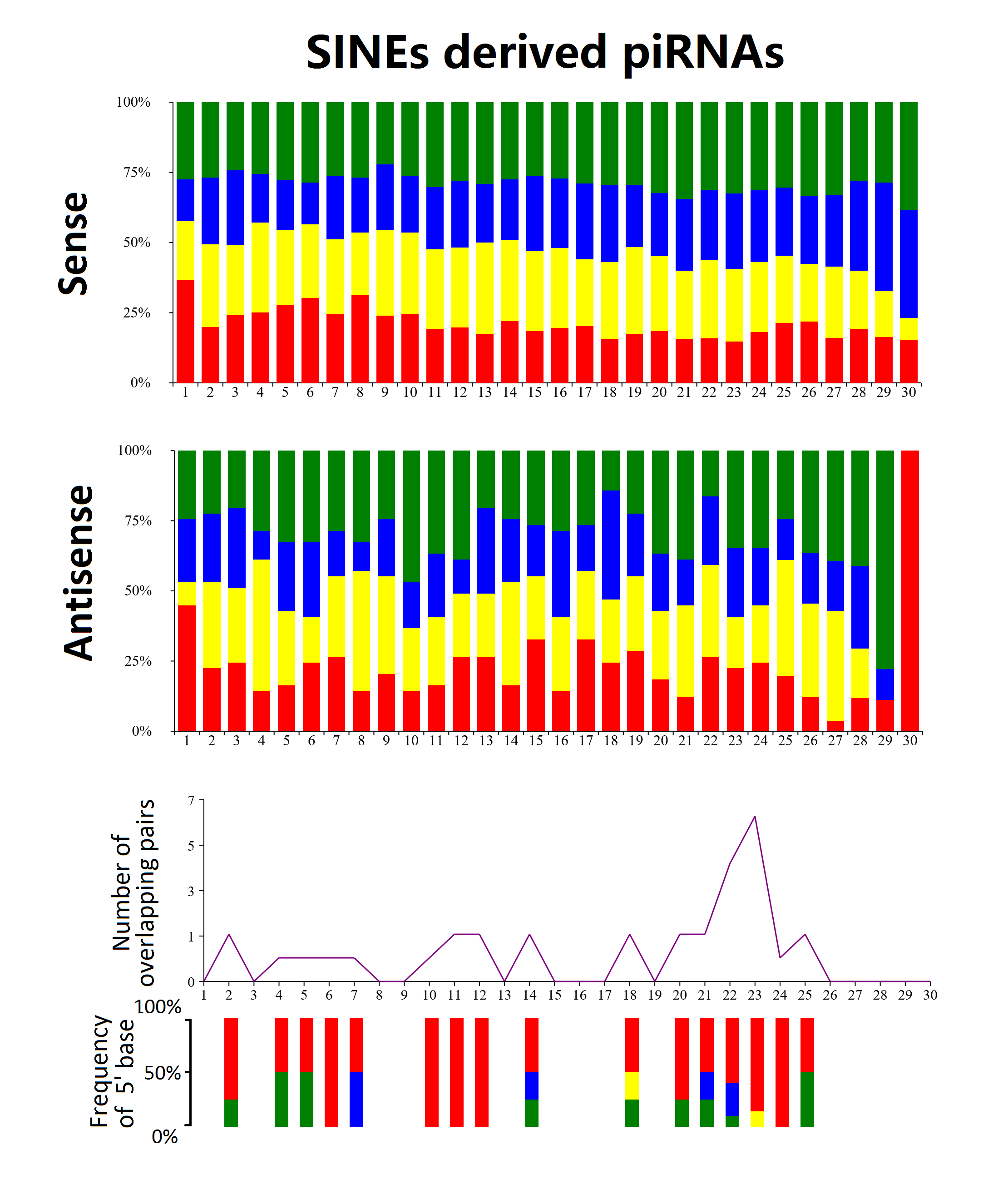

Supplement: Additional file 18: Figure S6. — Characterization of SINE-derived piRNAs. Base composition of SINE-derived piRNAs. The X-axis represents the nucleotide position relative to the 5′ ends of the piRNAs. The Y-axis represents the percentage of base bias. Lower pane: ping-pong pair analysis. The length of overlap is shown on the horizontal axes. Indicated above each axis is the number of possible overlapping pairs of small RNAs within a specified overlap size. Indicated below each axis is the relative frequency of the 5′ base identity for overlapping sequences. The colour code for bases is indicated in the centre box. (TIF 730 kb) [file 13071_2016_1815_MOESM18_ESM.tif]
